# Extra‐virgin olive oil enriched with lycopene: From industrial tomato by‐products to consumer

**DOI:** 10.1002/fsn3.4224

**Published:** 2024-05-22

**Authors:** Idoya Fernandez‐Pan, Sandra Horvitz, Francisco C. Ibañez, Cristina Arroqui, María José Beriain, Paloma Virseda

**Affiliations:** ^1^ IS‐Food Research Institute, Public University of Navarre Pamplona Spain

**Keywords:** carotenoid recovery, consumer acceptability, green solvent, high‐pressure processing, tomato waste

## Abstract

Lycopene is usually extracted from the by‐product of the tomato industry using organic solvents (OS) in combination with a physical technique. An emerging physical technique is high‐pressure processing (HPP). This study aims to find a method by applying a green solvent (edible vegetable oils) in an HPP‐assisted solid–liquid extraction. Three dosages of tomato by‐product (10%, 20%, and 40%, w/v) were tested using OS, sunflower oil (RSO), and extra‐virgin olive oil (EVOO). Lycopene recovery increased with the ratio of by‐product to oil, particularly when using EVOO. In another stage of the study, consumers evaluated EVOO that contained two doses of tomato by‐product (10% and 20%, w/v). Consumers preferred the EVOO from 10% tomato by‐product ratio over that with 20%. Additionally, 83.8% of consumers stated that enriched oil could be deemed beneficial for health. The proposed method considers the fundamental principles of the circular economy and practical industrial scenario to recover lycopene from tomato by‐product.

## Graphical abstract

Tomato lycopene recovery by edible vegetable oils as an alternative to organic solvents. Extraction facilitated by applying high‐hydrostatic pressures. Assessment of the acceptability of an olive oil enriched in lycopene.

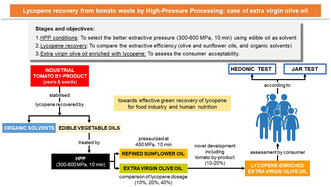

## 1 INTRODUCTION

The tomato processing industry generates a large volume of by‐products, namely seeds, pulp, and skin, which usually contain high levels of bioactive compounds including lycopene and other carotenoids. Lycopene, a carotenoid found in tomatoes and their processed derivatives, occurs in amounts ranging from 8.8 to 42 μg/g of wet weight and typically comprises up to 80%–90% of the total carotenoid fraction in ripe fruit (Rao et al., 2006).

The addition of tomato by‐products into traditional products was studied as a means of improving their nutritional value while reducing industrial wastes. In this sense, an increase in the fiber, polyphenols, and carotenoids content of gluten‐free (Betrouche et al., 2022) and wheat pasta (Padalino et al., 2017) enriched with tomato pomace was reported. As well, tomato by‐products were used as a source of functional components for the formulation of bread (Nour et al., 2015), bread and muffins (Mehta et al., 2018), and cream crackers (Nakov et al., 2022). These authors found that the addition of the tomato pomace enhanced the nutritional quality of the enriched products which also presented a good sensory quality.

Several methods can be employed to extract carotenoids from tomato by‐products, based on their physicochemical properties, biocompound profile and content, and the food matrix. In the case of lycopene, the process is conditioned by the hydrophobicity of this biocompound. Extraction is typically performed by combining polar and nonpolar organic solvents (OS), which in turn can be aided by enzymes (Catalkaya & Kahveci, 2019), ultrasound, and microwaves, among others. Even though this method provides cost‐effective basic processing operations, it involves large amounts of solvents, needs multiple cycles of extraction, recovery, and purification, and produces a significant volume of waste (Martins & Ferreira, 2017; Strati & Oreopoulou, 2014). Thus, different emerging technologies, such as ohmic heating (Coelho et al., 2023), pulsed electric fields (Pataro et al., 2020), ultrasound (Yadav et al., 2023), microwaves (Ho et al., 2015), and high‐pressure processing (HPP) (Briones‐Labarca et al., 2019) have been proposed as alternative methods for lycopene extraction. Indeed, HPP at 400–500 MPa has been shown to be effective in enhancing lycopene extraction from tomato pulp (Briones‐Labarca et al., 2019) and tomato paste waste (Xi, 2006a) by causing structural changes in the cells, the rupture of the tissues, and an increase in cells permeability and the diffusion of the solvents (Lara‐Abia et al., 2021). In comparison with solvent extraction, this method not only provided higher extraction yields, but also required shorter processing times (Xi, 2006b).

Additionally, edible vegetable oils acting as “green solvents” have been examined in the extraction of lycopene from commercially available tomatoes (Torres‐Valenzuela et al., 2020). Vegetable oils are of technological importance as they delay the oxidation and degradation processes of the extracted compounds by functioning as an oxygen barrier (Kunthakudee et al., 2020). Therefore, vegetable oil enriched with lycopene could be beneficial for human health and culinary technology. Specifically, olive oil has been proposed as a lipophilic and edible solvent to recover lycopene, as well as consuming the lycopene‐enriched olive oil for its health‐promoting benefits (da Silva et al., 2023; Eslami et al., 2023). However, consumers' acceptability studies for these novel products have not yet been conducted.

The hypothesis of this study was that the combination of HPP and an edible oil would enhance the recovery of lycopene from industrial tomato by‐products improving the extraction yield. Thus, the following objectives were proposed: (1) to evaluate the use of HPP to assist in an OS‐free extraction and diffusion of the remaining lycopene from the by‐products of the industrial production of tomato sauce; (2) to determine the appropriate conditions for achieving a lycopene‐enriched oil with culinary uses and healthy attributes accepted by consumers.

## 2 MATERIALS AND METHODS

The research was conducted in three stages: (i) characterization and stabilization of tomato by‐product, (ii) recovery of carotenoids and lycopene from tomato by‐product through an HPP‐assisted green solvent solid:liquid extraction procedure, and (iii) development of an edible oil enriched with lycopene from tomato by‐product and evaluation of acceptability to consumers.

### 2.1 Stage 1: Tomato by‐product characterization and stabilization

The tomato by‐product, gently provided by AN‐Conservas Dantza©, consisted of seeds and skins from the red pear tomato (Gladis variety). This fruit is a hybrid, elongated, and oblong in shape, fleshy, and very thin skinned. It is commonly used in the Navarre food industry to produce canned tomatoes. The skin is separated after blanching during the industrial production of tomato sauce. A total of 80 kg, corresponding to the autumn 2022 harvest and processing season, was used. Upon reception, the product, generated 24 h earlier and kept refrigerated in the industry, was immediately frozen at −20°C for preservation and storage throughout the entire experimental phase. A proximate composition analysis was conducted using official methods. The crude protein content of the samples was estimated by digestion at 390°C for 4 h (ISO 20483:2013), using a nitrogen–protein conversion factor of 6.25. The moisture content was evaluated after a drying time of 24 h at 130 ± 3°C (ISO 712:2009). The crude fat was quantified using the American Association of Cereal Chemists method (AACC, 2010). The ashes were estimated by incineration for 24 h at 525°C (ISO 2171:2007). Finally, the carbohydrate content was calculated from the mean values of the aforementioned parameters (Merrill & Watt, 1955).

The determination of the lycopene content in the tomato by‐product was carried out using a one‐step extraction with hexane, acetone, and ethanol in a 2:1:1 ratio additivated with 0.05% (v/v) of butylated hydroxytoluene (BHT, Merck KGaA) (Suwanaruang, 2016). Three samples containing 4% (w/v) of previously freeze‐dried by‐product (semi‐industrial freeze‐dryer LyoBeta 4PS, Telstar Spain, S.L.U.) and solvent were placed in an orbital shaker (Advanced Mini Shaker 15, VWR® International Eurolab S.L.) and kept under constant mechanical agitation at 33 rpm for 30 min at 25°C, while being protected from light. The samples were filtered and then 20% (v/v) of deionized water was added. After 5 min, the phases were decanted, and the nonpolar phase was collected for the quantification of the lycopene. The total lycopene was calculated by measuring the optical density at 503 nm using a MultiskanGo spectrophotometer (Thermo Fisher Scientific). A calibration line was set up using lycopene standard (tomato lycopene ≥ 98%, Cas. No. 502‐65‐8 Sigma Aldrich–Merck KGaA).

### 2.2 Stage 2: Recovery of carotenoids and lycopene by HPP‐assisted extraction with edible oils

An HPP‐assisted procedure was implemented for the recovery of carotenoids and lycopene from the tomato by‐product, using edible commercial vegetable oils as green solvents for the solid–liquid extraction. The type of edible oil (refined sunflower [RSO] and extra‐virgin olive [EVOO]), by‐product:oil ratio (10%, 20%, and 40% w/v) and pressurization treatment (300, 450, and 600 MPa for 10 min) were considered as the experimental design variables. The processing consisted of the following steps: For each corresponding solid:liquid ratio, the tomato by‐product and the edible oil, additivated with 0.05% (v/v) BHT, were brought into contact in polyethylene bags, which were sealed. Afterward, a Food‐Lab 900 equipment (Stansted Fluid Power Ltd.) was used to apply the HPP treatment. The bags were then immersed in a 45°C thermostatic bath for 45 min with constant agitation at 33 rpm. Finally, the vegetable oil was collected and kept in darkness until analyzed. The processing lasted 100 min from the contact of the by‐product with the oil to the analysis, and all the processes applied were carried out in triplicate. RSO (variety mix with a 24%–32% in oleic acid, Urzante SL) and EVOO (Arroniz variety, 79% in oleic acid, El Trujal Mendía S Coop.) acquired in a local supermarket were used as vegetable edible oils in all experimental tests and procedures. Both oils comply with the requirements of Spanish legislation (Royal Decree 308/1983, approving the Technical‐Sanitary Regulations for Edible Vegetable Oils).

Initially, a pressure screening was conducted to evaluate the general behavior of the system regarding carotenoids and lycopene recovery. The screening was performed at pressures from 300 to 600 MPa during 10 min, using samples containing 20% (w/v) of tomato by‐product. Following this, three different by‐product concentrations (10%, 20%, and 40% w/v) in each edible vegetable oil were tested for carotenoids and lycopene recovery at the selected pressure.

To determine the total carotenoids and lycopene content in the edible oils, the method proposed by Minguez‐Mosquera et al. (1991), based on hexane extraction and spectrophotometric determination in EVOO, was followed. This methodology allows simultaneous quantification of the chlorophyll fraction in the olive oil, which was also recorded, though it was not the focus of the study. Oil samples were also directly read at 300–750 nm to obtain their spectra profile. Standard regression lines were developed for quantification purposes from oil spiked with known quantities of standard lycopene.

### 2.3 Stage 3: Development of a lycopene‐enriched oil and evaluation of consumer acceptability

Following the same procedure exposed for stage 2, samples of EVOO enriched with three different tomato by‐product concentrations and a non‐enriched control were assessed in first instance by an internal panel of seven expert judges from the laboratory staff. A focus group session was held to discuss attributes such as aroma, color, bitterness, spiciness, and aftertaste. Additionally, the overall assessment and preference order were discussed. As a result of this preliminary test, the EVOO enriched with 10% and 20% tomato by‐product were selected for the overall consumer's test performance. The consumer's sensory analysis included three tests: (i) an acceptability test using a 9‐point hedonic scale (Lawless & Heymann, 2010), (ii) an acceptability test using an ideal point scale (Just‐About‐Right, JAR) (Rothman, 2007), and (iii) a preference ranking test (ISO 8587:2006).

A panel of 80 regular consumers of EVOO was recruited, consisting of 54 women and 26 men. In a standardized room (ISO 8589:2007), two coded samples were presented simultaneously and tested in two ways: First, 10 mL of each oil sample were presented in a 20 mL transparent glass, and second, each oil was presented in a 2‐mL pipette that the consumer had to pour over a piece of bread toast. Between samples, consumers were instructed to wipe their mouths with a slice of golden apple, which was presented cut into 4‐mm thick pieces. Before the test, consumers were informed about and consented to the confidentiality of their personal data processing (Regulation EU 2016/679 of the European Parliament and of the Council, of April 27, 2016), as well as the terms and conditions of the sensory evaluation.

### 2.4 Statistical analyses

The data from stages 1 and 2 underwent a one‐way ANOVA analysis (α = 0.05) using the Statgraphics 18.0 software (Statgraphics Technologies, Inc.). When significant differences were detected, mean treatments were compared using Tukey's test. Stage 3 data were subjected to a nonparametric analysis using the Mann–Whitney *U* test. Additionally, a penalty analysis for the JAR test was applied.

## 3 RESULTS AND DISCUSSION

### 3.1 Tomato by‐product characterization

The received tomato by‐product was formed of skins and seeds and presented a proximal composition made up of 84.95% moisture, 3.33% protein, 1.65% fat, 0.86% ashes, and 9.21% total carbohydrates. The lycopene content in the by‐product was extracted conventionally using OS (2:1:1 hexane, acetone, ethanol) and resulted in 1174.27 ± 43.13 mg lycopene/kg DW by‐product. These values were similar to those reported by Szabo et al. (2022) (1184.15 ± 2.20 lycopene/kg DW of tomato by‐product) and Kehili et al. (2019) (1244 ± 30.41 lycopene/kg DW of tomato by‐product). Many research works reported also higher and lower lycopene contents in tomato by‐products and these differences could be due to differences in tomato cultivars and maturity stage, moisture content, pomace composition, and extraction methods (Tran et al., 2023).

### 3.2 Recovery of carotenoids and lycopene by HPP‐assisted extraction with edible oils

The 350–700 nm spectra profiles of lycopene standard in the hexane:acetone:ethanol OS, EVOO, and RSO presented the characteristic three peaks of the molecule. In the OS and RSO cases, and in good agreement with the literature, the three maxima absorbance peaks were in 444, 472, and 503 nm (Anthon & Barrett, 2007). The EVOO, as expected, exhibited its own absorption spectrum with wavelengths of maxima absorbance peaks linked to the carotenoid and chlorophyll fractions, associated with those needed for data processing. These wavelengths were identified and set at 414, 482, and 670 nm when the oil was directly read, and at 474, 508, and 670 nm in the case of a previous hexane extraction (indirect reading). The selected wavelengths are very close to those proposed by Minguez‐Mosquera et al. (1991) at 472, 503, and 670 nm. Appropriate linear regression coefficients were obtained by establishing standard curves for quantifying lycopene in the edible vegetable oils both via direct and indirect methods. A selection of spectra profiles and regression lines are shown in Figures S1–S4 and Table S1. It should be noted that there are various methods for extracting and quantifying lycopene in literature, with spectrophotometry being the most common. This involves taking readings after extracting with a solvent mixture containing hexane, acetone, and ethanol at approximately 480 and 503 nm wavelengths (Poojary & Passamonti, 2015; Popescu et al., 2022; Silva et al., 2019; Zuorro, 2020). These methodological proposals may not be practical for the food industry setting, which often requires the use of rapid identification procedures that provide immediate and economically feasible outputs. In our case, although all spectra profiles obtained from direct reading were similar, it was detected that the baseline drifted upward for the oil samples after the by‐product contact, which increased the absorbance. This may be due to the presence of water, different types, ratios, and mixture of carotenoids, and other released substances from the by‐product absorbing in the same region. For this reason, the indirect method has been chosen for quantitative purposes. Therefore, the one step hexane extraction method proposed by Minguez‐Mosquera et al. (1991) was selected in this work. That is, the indirect reading method for the determination of carotenoids and lycopene as well as chlorophylls present in the RSO and EVOO oils after the enrichment process from by‐products of the tomato industry.

**FIGURE 1:**
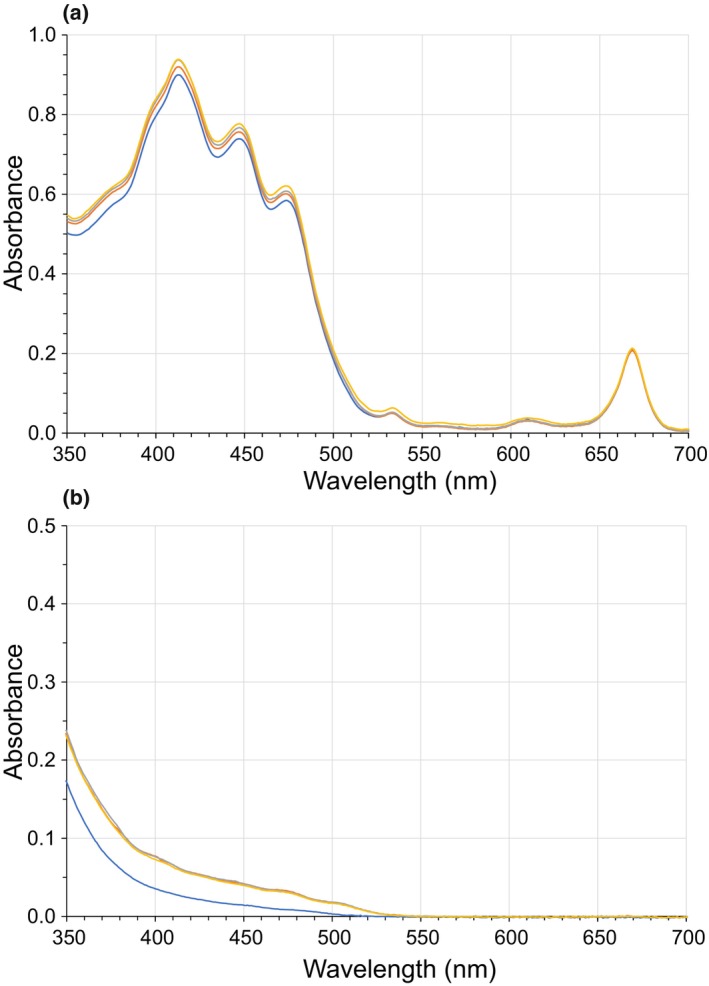
Absorption spectra of extra‐virgin olive oil (a) and refined sunflower oil (b) after different HPP‐assisted 20% (w/v) by‐product in oil enrichment processing (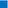: unpressurized; 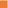: 300 MPa; 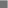: 450 MPa; 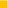: 600 MPa).

Regarding the intensity of the pressurization process to be fixed for the HPP extraction assistance process, a 20% (w/v) of by‐product in RSO and EVOO was treated under 300, 450, and 600 MPa for 10 min with previous confirmation that pressurization of standard lycopene added EVOO and RSO did not modify the absorption spectra profile nor the selected maxima peaks wavelength. No differences were detected for the recovery of carotenoids fraction and lycopene at the different pressurization treatments applied (Figure 1). Qiu et al. (2006) pointed out that the stability and isomerization of lycopene from tomato purée by HPP depended on the processing conditions, and solvent or food matrix. They reported that 400 MPa retained the maximal stability of lycopene in solution while the highest stability of lycopene was found when tomato purée was pressurized at 500 MPa and cold stored. Taking into account the results and the range of pressures referred to by Qiu et al. (2006), the intermediate treatment at 450 MPa/10 min was chosen to continue with the study.

At this point, independent 10%, 20%, and 40% w/v by‐product:oil ratio samples were treated and, as listed in Tables 1 and 2, it can be stated that the higher the ratio of by‐product:oil, the greater the enrichment of RSO and EVOO in lycopene (mg lycopene/L oil), but the lower the extraction yield (mg lycopene/kg by‐product). This may be because for a higher by‐product:oil ratio, both the migration gradient and the contact surface of the skins and seeds with the oil are lower, slowing down the extraction process, that has been conducted for fixed extraction times and agitation in all cases. Moreover, it is important to note that both the lycopene recovery yield and the oil enrichment were higher for EVOO than for RSO. Thus, the lycopene recovery yields for EVOO ranged from 50% to 80%, compared to 26%–41% for RSO. This resulted in an enrichment of the oils ranging from 14 to 32 mg lycopene/L for EVOO and 6–16 mg lycopene/L for RSO.

**TABLE 1:** Lycopene recovery from different by‐product:oil ratios.

Oil type	By‐product:oil ratio (w/v)	Lycopene recovery (mg/kg by‐product)	Recovery yield (%)
EVOO	10	142.40 ± 7.40^c^	80.84
	20	113.29 ± 3.21^b^	64.31
	40	90.13 ± 4.82^a^	51.17
RSO	10	73.55 ± 9.06^c^	41.75
	20	58.98 ± 5.06^b^	33.48
	40	46.63 ± 2.48^a^	26.47

*Note*: Values are expressed as mean ± standard deviation (*n* = 3). Different letters indicate significant differences under the Tukey's test (*p* < .05).

Abbreviations: EVOO, extra‐virgin olive oil; RSO, refined sunflower oil.

**TABLE 2:** Lycopene enrichment of extra‐virgin olive oil (EVOO) and refined sunflower oil (RSO) from different by‐product:oil ratios.

Oil type	By‐product:oil ratio (w/v)	Lycopene content in oil (mg/L)
EVOO	10	14.03 ± 0.67^a^
	20	20.44 ± 0.60^b^
	40	32.06 ± 1.61^c^
RSO	10	6.83 ± 0.76^a^
	20	10.62 ± 0.89^b^
	40	16.91 ± 0.97^c^

*Note*: Values are expressed as mean ± standard deviation (*n* = 3). Different letters indicate significant differences under the Tukey's test (*p* < .05).

### 3.3 Consumers assessment of EVOO enriched with lycopene

The resulting oils were evaluated internally by a panel of seven judges who assessed the gain in aroma, color, and flavor from the tomato by‐product, as well as the lycopene content of each sample. These characteristics were valued positively in the EVOO containing the two highest by‐product:oil ratio, and in no case for RSO, since it is generally intended for thermally aggressive culinary uses such as frying. Such techniques would result in the thermal destruction of lycopene, and furthermore, the small amounts of bioactive obtained would not be considered beneficial for health nor a viable economic antioxidant additive solution. Therefore, a minimum enrichment level of 20 mg/L was positively valued for raw consumption, and a consumer acceptability test was carried out on the EVOO from the enrichment process using two different by‐product ratios.

The sensory analysis was conducted with a total of 80 individuals, whose main characteristics are described in Table 3. The sensory panelists evaluated the lycopene‐enriched EVOO obtained from samples containing 10% and 20% tomato by‐product, first directly and then poured on a bread toast.

**TABLE 3:** Main characteristics of the consumers' panel participating in the sensory analysis of the lycopene‐enriched extra‐virgin olive oil.

Variable	Categories	Percent
Gender	Female	67.50
	Male	32.50
Age	18–30 years	56.30
	30–50 years	16.20
	>50 years	27.50
EVOO consumption habits	Regular consumers	95.00
	Nonregular consumers	5.00
EVOO consumption frequency	Daily	72.50
	3 or more times/week	13.75
	1–2 times/week	7.50
	1–2 times/month	2.50
	Occasionally	3.75
Positive assessment toward bioactive‐enriched products	Yes	76.30
	No	23.70
Consumption of bioactive‐enriched products	Yes	35.00
	No	65.00

**FIGURE 2:**
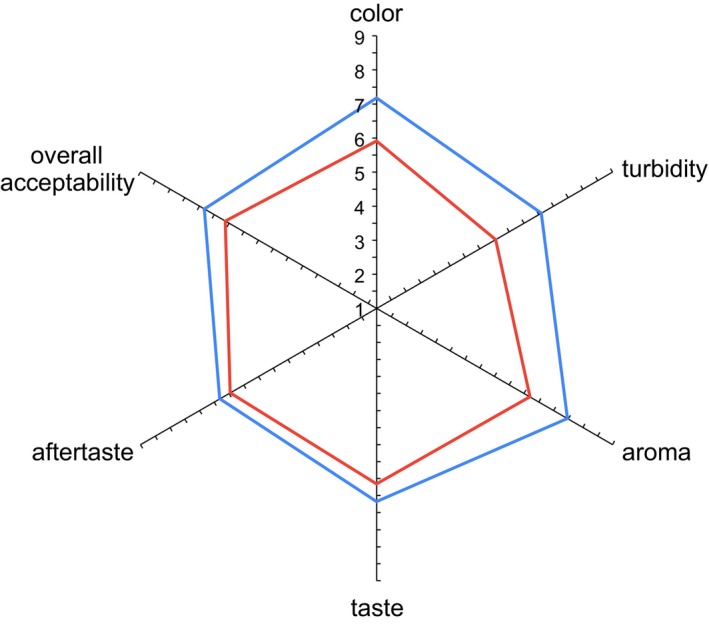
Hedonic profile of olive oil containing tomato by‐product (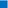: 10% w/v; 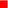 20% w/v).

Figure 2 shows the scores for the sensory attributes in the hedonic test when the oil was directly evaluated. As it can be seen, the EVOO containing the lowest lycopene content received significantly higher punctuations in all the evaluated attributes. These samples received scores ranging from 6 (“I like it a little”) to 7 (“I quite like it”) for the turbidity, taste, aftertaste, and overall acceptability. It is important to note that aroma and color scores were above 7, which corresponded to the “I like it very much” category. The panelists showed a clear preference for a greener rather than a reddish color. This preference aligns with the findings of Vázquez‐Araújo et al. (2015) who observed that Spanish consumers tend to favor dark green olive oils due to their association with high quality and superior flavor. On the other hand, most of the scores of the EVOO sample with the highest lycopene content were in the 5 (neither like nor dislike) to 6 (“I like it a little”) interval. However, even though these samples were perceived as “too cloudy,” the overall acceptability scored above 6 (“I like it a little” to “I quite like it”).

**FIGURE 3:**
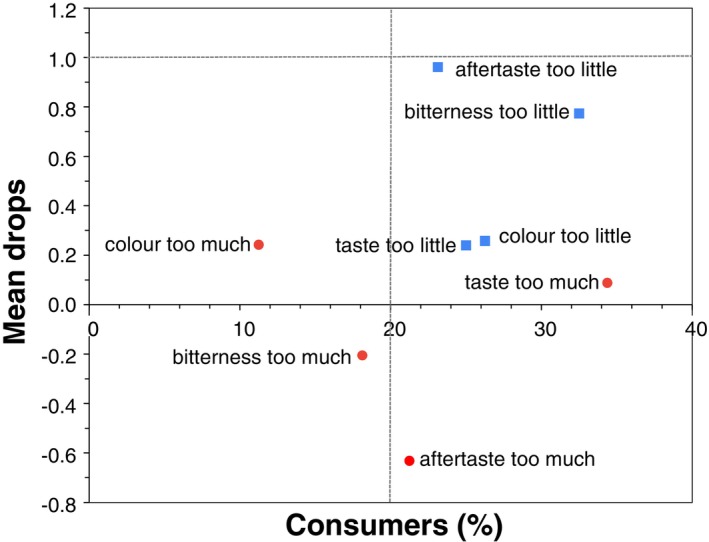
Penalty test from JAR results for olive oils enriched with tomato by‐product (10% and 20% w/v). (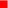 more intensity; 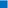 less intensity). The gray dotted lines represent the cut‐offs for consumers (20%) and mean drops (1.0).

No significant differences were observed between the samples for any of the attributes when the oils were evaluated on the toast. The scores for all attributes ranged between 6.1 and 6.7 in this test, with flavor and acceptability receiving the highest values. The results for the JAR test are shown in Figure 3.

**FIGURE 4:**
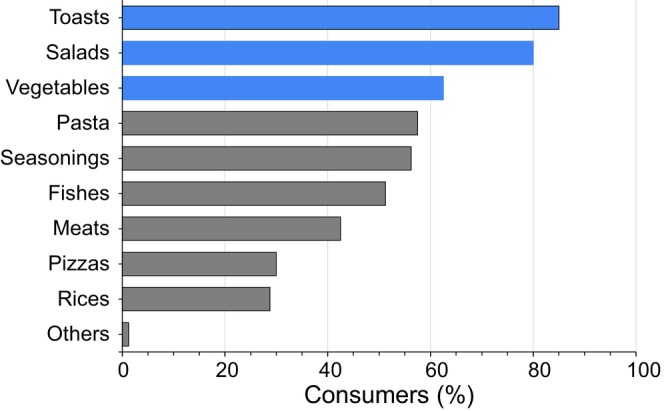
Potential uses of lycopene‐enriched olive oil based on consumer survey (applications above consumer 60% are shown in blue).

According to the survey results (Figure 4), consumers favored using this type of oil for toasts, salads, and vegetables. In addition, 50% of the panelists valued it as a gourmet product, 83.8% associated it with health, and 75% positively valued that it was produced from by‐products. According to the literature, consumers associate the intake of olive oil with beneficial effects on health (Ilak‐Peršurić & Težak‐Damijanić, 2021; Pichierri et al., 2020; Santosa et al., 2013), while the country of origin, the region of production, and protected designation of geographical origin labels are important factors when choosing a specific product for consumption (Ilak‐Peršurić, 2020; Perito et al., 2019; Spognardi et al., 2021).

## 4 CONCLUSIONS

An HPP‐assisted solid–liquid extraction processing has been proposed as a “green process” for the lycopene enrichment of RSO and EVOO. The process allows the direct extraction and diffusion of the lycopene and carotenoids from the tomato by‐product to the vegetable edible oils. Due to the higher recovery yield and oil enrichment for the same by‐product:oil ratio, the EVOO resulted in the most interesting proposal. Furthermore, the consumers' acceptability of the lycopene‐enriched EVOO supports and validates the underlying concept. The proposed technique allowed the revalorization of the industrial tomato by‐product based on a sustainable processing, while enhancing the health‐related properties of the olive oil.

## AUTHOR CONTRIBUTIONS

**Idoya Fernandez‐Pan:** Data curation (equal); formal analysis (equal); investigation (equal); methodology (equal); visualization (equal); writing – original draft (equal); writing – review and editing (equal). **Sandra Horvitz:** Formal analysis (equal); visualization (equal); writing – original draft (equal); writing – review and editing (equal). **Francisco C. Ibañez:** Data curation (equal); formal analysis (equal); investigation (equal); visualization (equal); writing – original draft (equal); writing – review and editing (equal). **Cristina Arroqui:** Formal analysis (equal); investigation (equal); visualization (equal); writing – review and editing (equal). **María José Beriain:** Conceptualization (equal); formal analysis (equal); funding acquisition (equal); investigation (equal); methodology (equal); project administration (equal); writing – review and editing (equal). **Paloma Virseda:** Conceptualization (equal); formal analysis (equal); funding acquisition (equal); investigation (equal); methodology (equal); project administration (equal); visualization (equal); writing – review and editing (equal).

## FUNDING INFORMATION

This project has been funded by the Government of Navarre through the programme for the Implementation of Strategic R&D Projects for the period 2021‐2024. This funding is part of Navarre´s contribution to the AGROALNEXT Complementary Agri‐Food Plan, which is included in Component 17 Investment 1 of the Recovery, Transformation and Resilience Plan (ALISSEC project 0011‐1411‐2021).

## CONFLICT OF INTEREST STATEMENT

The authors report there are no competing interests to declare.

## ETHICS STATEMENT

Verbal informed consent was obtained from all participants involved in this study.

## Supporting information


Data S1:

## Data Availability

Research data are available within the article.

## References

[fsn34224-bib-0044] AACC . (2010). Crude fat in wheat, corn, and soy flour, feeds, and mixed feeds (30‐25.01). Cereals & Grains Association. 10.1094/AACCIntMethod-30-25.01

[fsn34224-bib-0001] Anthon, G. , & Barrett, D. M. (2007). Standardization of a rapid spectrophotometric method for lycopene analysis. Acta Horticulturae, 758, 111–128. 10.17660/ActaHortic.2007.758.12

[fsn34224-bib-0002] Betrouche, A. , Estivi, L. , Colombo, D. , Pasini, G. , Benatallah, L. , Brandolini, A. , & Hidalgo, A. (2022). Antioxidant properties of gluten‐free pasta enriched with vegetable by‐products. Molecules, 27(24), 8993. 10.3390/molecules27248993 36558126 PMC9784952

[fsn34224-bib-0003] Briones‐Labarca, V. , Giovagnoli‐Vicuña, C. , & Cañas‐Sarazúa, R. (2019). Optimization of extraction yield, flavonoids and lycopene from tomato pulp by high hydrostatic pressure‐assisted extraction. Food Chemistry, 278, 751–759. 10.1016/j.foodchem.2018.11.106 30583438

[fsn34224-bib-0004] Catalkaya, G. , & Kahveci, D. (2019). Optimization of enzyme assisted extraction of lycopene from industrial tomato waste. Separation and Purification Technology, 219, 55–63. 10.1016/j.seppur.2019.03.006

[fsn34224-bib-0005] Coelho, M. C. , Ghalamara, S. , Campos, D. , Ribeiro, T. B. , Pereira, R. , Rodrigues, A. S. , Teixeira, J. A. , & Pintado, M. (2023). Tomato processing by‐products valorisation through ohmic heating approach. Food, 12(4), 818. 10.3390/foods12040818 PMC9957376 36832895

[fsn34224-bib-0006] da Silva, P. B. V. , Brenelli, L. B. , & Mariutti, L. R. B. (2023). Waste and by‐products as sources of lycopene, phytoene, and phytofluene – Integrative review with bibliometric analysis. Foodservice Research International, 169, 112838. 10.1016/j.foodres.2023.112838 37254412

[fsn34224-bib-0007] Eslami, E. , Carpentieri, S. , Pataro, G. , & Ferrari, G. (2023). A comprehensive overview of tomato processing by‐product valorization by conventional methods versus emerging technologies. Food, 12(1), 166. 10.3390/foods12010166 PMC9818577 36613382

[fsn34224-bib-0008] Ho, K. K. H. Y. , Ferruzzi, M. G. , Liceaga, A. M. , & San Martín‐González, M. F. (2015). Microwave‐assisted extraction of lycopene in tomato peels: Effect of extraction conditions on all‐trans and cis‐isomer yields. LWT – Food Science and Technology, 62(1), 160–168. 10.1016/j.lwt.2014.12.061

[fsn34224-bib-0009] Ilak‐Peršurić, A. S. (2020). Segmenting olive oil consumers based on consumption and preferences toward extrinsic, intrinsic and sensorial attributes of olive oil. Sustainability, 12(16), 6379. 10.3390/su12166379

[fsn34224-bib-0010] Ilak‐Peršurić, A. S. , & Težak‐Damijanić, A. (2021). Connections between healthy behaviour, perception of olive oil health benefits, and olive oil consumption motives. Sustainability, 13(14), 7630. 10.3390/su13147630

[fsn34224-bib-0011] Kehili, M. , Sayadi, S. , Frikha, F. , Zammel, A. , & Allouche, N. (2019). Optimization of lycopene extraction from tomato peels industrial by‐product using maceration in refined olive oil. Food and Bioproducts Processing, 117, 321–328. 10.1016/j.fbp.2019.08.004

[fsn34224-bib-0012] Kunthakudee, N. , Sunsandee, N. , Chutvirasakul, B. , & Ramakul, P. (2020). Extraction of lycopene from tomato with environmentally benign solvents: Box‐Behnken design and optimization. Chemical Engineering Communications, 207(4), 574–583. 10.1080/00986445.2019.1610882

[fsn34224-bib-0013] Lara‐Abia, S. , Welti‐Chanes, J. , & Cano, M. P. (2021). Effect of high hydrostatic pressure on the extractability and bioaccessibility of carotenoids and their esters from papaya (*Carica papaya* L.) and its impact on tissue microstructure. Food, 10(10), 2435. 10.3390/foods10102435 PMC8535580 34681484

[fsn34224-bib-0014] Lawless, H. T. , & Heymann, H. (2010). Sensory evaluation of food: Principles and practices. Springer.

[fsn34224-bib-0015] Martins, N. , & Ferreira, I. C. F. R. (2017). Wastes and by‐products: Upcoming sources of carotenoids for biotechnological purposes and health‐related applications. Trends in Food Science and Technology, 62, 33–48. 10.1016/j.tifs.2017.01.014

[fsn34224-bib-0016] Mehta, D. , Prasad, P. , Sangwan, R. S. , & Yadav, S. K. (2018). Tomato processing byproduct valorization in bread and muffin: Improvement in physicochemical properties and shelf life stability. Journal of Food Science and Technology, 55(7), 2560–2568. 10.1007/s13197-018-3176-0 30042572 PMC6033793

[fsn34224-bib-0017] Merrill, A. L. , & Watt, B. K. (1955). Energy value of foods: Basis and derivation (p. 74). United States Department of Agriculture.

[fsn34224-bib-0018] Minguez‐Mosquera, M. I. , Rejano‐Navarro, L. , Gandul‐Rojas, B. , SanchezGomez, A. H. , & Garrido‐Fernandez, J. (1991). Color‐pigment correlation in virgin olive oil. Journal of the American Oil Chemists' Society, 68(5), 332–336. 10.1007/BF02657688

[fsn34224-bib-0019] Nakov, G. , Brandolini, A. , Estivi, L. , Bertuglia, K. , Ivanova, N. , Jukić, M. , Komlenić, D. K. , Lukinac, J. , & Hidalgo, A. (2022). Effect of tomato pomace addition on chemical, technological, nutritional, and sensorial properties of cream crackers. Antioxidants, 11(11), 2087. 10.3390/antiox11112087 36358460 PMC9686889

[fsn34224-bib-0020] Nour, V. , Ionica, M. E. , & Trandafir, I. (2015). Bread enriched in lycopene and other bioactive compounds by addition of dry tomato waste. Journal of Food Science and Technology, 52(12), 8260–8267. 10.1007/s13197-015-1934-9 26604402 PMC4648879

[fsn34224-bib-0021] Padalino, L. , Conte, A. , Lecce, L. , Likyova, D. , Sicari, V. , Pellicanò, T. M. , Poiana, M. , & Del Nobile, M. A. (2017). Functional pasta with tomato by‐product as a source of antioxidant compounds and dietary fibre. Czech Journal of Food Science, 35(1), 48–56. 10.17221/171/2016-CJFS

[fsn34224-bib-0022] Pataro, G. , Carullo, D. , Falcone, M. , & Ferrari, G. (2020). Recovery of lycopene from industrially derived tomato processing by‐products by pulsed electric fields‐assisted extraction. Innovative Food Science & Emerging Technologies, 63, 102369. 10.1016/j.ifset.2020.102369

[fsn34224-bib-0023] Perito, M. A. , Sacchetti, G. , Di Mattia, C. D. , Chiodo, E. , Pittia, P. , Saguy, I. S. , & Cohen, E. (2019). Buy local! Familiarity and preferences for extra virgin olive oil of Italian consumers. Journal of Food Products Marketing, 25(4), 462–477. 10.1080/10454446.2019.1582395

[fsn34224-bib-0024] Pichierri, M. , Peluso, A. M. , Pino, G. , & Guido, G. (2020). Communicating the health value of extra‐virgin olive oil: An investigation of consumers' responses to health claims. British Food Journal, 123(2), 492–508. 10.1108/BFJ-03-2020-0198

[fsn34224-bib-0025] Poojary, M. M. , & Passamonti, P. (2015). Extraction of lycopene from tomato processing waste: Kinetics and modelling. Food Chemistry, 173, 943–950. 10.1016/j.foodchem.2014.10.127 25466110

[fsn34224-bib-0026] Popescu, M. , Iancu, P. , Plesu, V. , & Bildea, C. S. (2022). Carotenoids recovery enhancement by supercritical CO_2_ extraction from tomato using seed oils as modifiers. Processes, 10(12), 2656. 10.3390/pr10122656

[fsn34224-bib-0027] Qiu, W. , Jiang, H. , Wang, H. , & Gao, Y. (2006). Effect of high hydrostatic pressure on lycopene stability. Food Chemistry, 97(3), 516–523. 10.1016/j.foodchem.2005.05.032

[fsn34224-bib-0028] Rao, A. V. , Ray, M. R. , & Rao, L. G. (2006). Lycopene. Advances in Food and Nutrition Research, 51, 99–164. 17011475 10.1016/S1043-4526(06)51002-2

[fsn34224-bib-0029] Rothman, L. (2007). The use of just‐about‐right (JAR) scales in food product development and reformulation. In H. MacFie (Ed.), Consumer‐led food product development (pp. 407–433). Woodhead Publishing.

[fsn34224-bib-0030] Santosa, M. , Clow, E. J. , Sturzenberger, N. D. , & Guinard, J.‐X. (2013). Knowledge, beliefs, habits and attitudes of California consumers regarding extra virgin olive oil. Foodservice Research International, 54(2), 2104–2111. 10.1016/j.foodres.2013.07.051

[fsn34224-bib-0031] Silva, Y. P. A. , Borba, B. C. , Pereira, V. A. , Reis, M. G. , Caliari, M. , Brooks, M. S.‐L. , & Ferreira, T. A. P. C. (2019). Characterization of tomato processing by‐product for use as a potential functional food ingredient: Nutritional composition, antioxidant activity and bioactive compounds. International Journal of Food Science and Nutrition, 70(2), 150–160. 10.1080/09637486.2018.1489530 30014726

[fsn34224-bib-0033] Spognardi, S. , Vistocco, D. , Cappelli, L. , & Papetti, P. (2021). Impact of organic and “protected designation of origin” labels in the perception of olive oil sensory quality. British Food Journal, 123(8), 2641–2669. 10.1108/BFJ-07-2020-0596

[fsn34224-bib-0034] Strati, I. F. , & Oreopoulou, V. (2014). Recovery of carotenoids from tomato processing by‐products – A review. Food Research International, 65, 311–321. 10.1016/j.foodres.2014.09.032

[fsn34224-bib-0035] Suwanaruang, T. (2016). Analyzing lycopene content in fruits. Agriculture and Agricultural Science Procedia, 11, 46–48. 10.1016/j.aaspro.2016.12.008

[fsn34224-bib-0036] Szabo, K. , Teleky, B.‐E. , Ranga, F. , Roman, I. , Khaoula, H. , Boudaya, E. , Ltaief, A. B. , Aouani, W. , Thiamrat, M. , & Vodnar, D. C. (2022). Carotenoid recovery from tomato processing by‐products through green chemistry. Molecules, 27(12), 3771. 10.3390/molecules27123771 35744898 PMC9231286

[fsn34224-bib-0037] Torres‐Valenzuela, L. S. , Ballesteros‐Gómez, A. , & Rubio, S. (2020). Green solvents for the extraction of high added‐value compounds from agri‐food waste. Food Engineering Reviews, 12(1), 83–100. 10.1007/s12393-019-09206-y

[fsn34224-bib-0038] Tran, D. T. , Nguyen, L. T. H. , Nguyen, C. N. , Hertog, M. L. A. T. M. , Nicolaï, B. , & Picha, D. (2023). Optimization of lycopene extraction from tomato pomace and effect of extract on oxidative stability of peanut oil. Polish Journal of Food and Nutrition Sciences, 73(3), 205–213. 10.31883/pjfns/168233

[fsn34224-bib-0039] Vázquez‐Araújo, L. , Adhikari, K. , Chambers, E. , Chambers, D. , & Carbonell‐Barrachina, A. (2015). Cross‐cultural perception of six commercial olive oils: A study with Spanish and US consumers. Food Science and Technology International, 21(6), 454–466. 10.1177/1082013214543806 25028154

[fsn34224-bib-0040] Xi, J. (2006a). Application of high hydrostatic pressure processing of food to extracting lycopene from tomato paste waste. High Pressure Research, 26(1), 33–41. 10.1080/08957950600608741

[fsn34224-bib-0041] Xi, J. (2006b). Effect of high pressure processing on the extraction of lycopene in tomato paste waste. Chemical Engineering & Technology, 29(6), 736–739. 10.1002/ceat.200600024

[fsn34224-bib-0042] Yadav, R. D. , Khanpit, V. V. , Dhamole, P. B. , & Mandavgane, S. A. (2023). Integrated ultrasound‐surfactant assisted extraction of lycopene from tomato peels. Chemical Engineering and Processing: Process Intensification, 191, 109474. 10.1016/j.cep.2023.109474

[fsn34224-bib-0043] Zuorro, A. (2020). Enhanced lycopene extraction from tomato peels by optimized mixed‐polarity solvent mixtures. Molecules, 25(9), 2038. 10.3390/molecules25092038 32349412 PMC7248986

