# Supplementary material for: Extra‐virgin olive oil enriched with lycopene: From industrial tomato by‐products to consumer
Source: Food Sci Nutr. 2024 May 22;12(8):5815–23. doi: 10.1002/fsn3.4224 (PMC11317748; doi:10.1002/fsn3.4224)
Supplement: Supplementary file 1 — Data S1: [file FSN3-12-5815-s001.docx]

**Supporting information**

TABLE S1**.** Regression lines for the calibration curves of lycopene content according to reading method (DM: direct method, after olive oil extraction; IM: indirect method, after hexane extraction).

| **Method** | **Wavelength** (nm) | **Regression line** | ***r^2^*** |
| --- | --- | --- | --- |
| DM | 414 | A = 0.0023 x [lycopene, mg/L] + 1.8907 | 0.9931 |
|  | 482 | A = 0.0047 x [lycopene, mg/L] + 1.2548 | 0.9373 |
|  | 670 | A = 0.0015 x [lycopene, mg/L] + 0.5379 | 0.9804 |
| IM | 474 | A = 0.0033 x [lycopene, mg/L] + 0.4573 | 0.9861 |
|  | 508 | A = 0.0030 x [lycopene, mg/L] + 0.125 | 0.9885 |
|  | 670 | A = 0.0005 x [lycopene, mg/L] + 0.2141 | 0.9739 |

FIGURE. S1. Absorption spectra profile of extra virgin olive oil samples (n = 3) measured by direct method (◼ control; ◼ 10% tomato by-product; ◼ 20% tomato by-product; ◼ 40% tomato by-product).

FIGURE S2. Absorption spectra profile of refined sunflower oil samples (n = 3) measured by direct method (◼ control; ◼ 10% tomato by-product; ◼ 20% tomato by-product; ◼ 40% tomato by-product).

FIGURE S3. Absorption spectra profile of extra virgin olive oil samples (n = 3) measured by indirect method (◼ control; ◼ 10% tomato by-product; ◼ 20% tomato by-product; ◼ 40% tomato by-product).

FIGURE S4. Absorption spectra profile of refined sunflower oil samples (n = 3) measured by indirect method (◼ control; ◼ 10% tomato by-product; ◼ 20% tomato by-product; ◼ 40% tomato by-product).
